# Differences in the mechanism of type 1 and type 2 diabetes-induced skin dryness by using model mice

**DOI:** 10.7150/ijms.50764

**Published:** 2021-01-01

**Authors:** Tsuneki Horikawa, Keiichi Hiramoto, Kenji Goto, Hidehisa Sekijima, Kazuya Ooi

**Affiliations:** 1 Department of Pharmaceutical Sciences, Suzuka University of Medical Science, Suzuka, Japan.; 2 Research Laboratories, Nichinichi Pharmaceutical Co., Ltd., Iga, Japan.; 3 Department of Forensic Medicine and Sciences, Graduate School of Medicine, Mie University, Tsu, Japan.

**Keywords:** Diabetes, STZ, KK-Ay/TaJcl mice, Skin dryness, Collagen, Hyaluronic acid

## Abstract

Diabetes induces dry skin that may cause infective diseases. In this study, we aimed to clarify the mechanism of diabetes-induced skin dryness in animal models. We also examined the difference in the mechanism of skin dryness in type 1 and type 2 diabetes. We examined skin dryness in type 1 diabetes model mice (streptozotocin [STZ] induction), non-obesity type 2 diabetes model mice (newborn STZ injection), and obesity type 2 diabetes model mice (KK-Ay/TaJcl). An increase in transepidermal water loss was observed in the type 1 diabetes model mice, and reduced skin hydration was observed in the type 2 diabetes model mice. In the type 1 diabetes model mice, an increase in advanced glycation end products and matrix metalloproteinase-9 led to a decline in collagen IV level, inducing skin dryness. In the obesity type 2 diabetes model mice, an increase in the release of histamine and hyaluronidase by mast cells resulted in a decline in the level of hyaluronic acid, inducing skin dryness. However, in the non-obesity type 2 diabetes model mice, the main factors of skin dryness could not be clearly identified. Nevertheless, inflammatory cytokine levels increased. We hypothesize that inflammatory cytokines disrupt the collagen of the skin. Diabetes caused skin dryness in each mouse model, and the mechanism of skin dryness differed by diabetes type.

## Introduction

The frequency of diabetic genesis is increasing, causing health impairment by nephropathy, retinopathy and neurosis. Diabetes not only leads to cardiovascular problems but also to the evolution of arteriosclerosis.

Diabetes is classified into type 1 (insulin-dependent diabetes) and type 2 (non-insulin-dependent diabetes) diabetes. Type 1 diabetes is an autoimmune disease, and symptoms develop when the B cell of the pancreas are destroyed. The cytotoxicity to the islet cell by T-lymphocytes has been proven in animal models 1. Furthermore, type 1 diabetes is considered to be an immunopathy in humans and animal models because of the inhibition of pancreatic B cells by immunosuppression 2. The onset of type 2 diabetes is influenced by genetic and environmental factors. In type 2 diabetes, the blood glucose level becomes chronically high because of the deficit in insulin functions. The deficit in functions of insulin indicates a decline in insulin secretion (insulin secretion deficiency) or the case where the insulin function is adversely affected (insulin resistance). Oxidative stress is one of the factors that have a role in causing a deficiency of insulin secretion 3. Pancreatic B cell, an insulin secretory cell, has particularly very little intercellular superoxide dismutase and is, therefore, weak against oxidative stress. The diabetic condition occurs during chronic hyperglycemia, in which excess glucose combines with a protein to form a glycated protein, causing oxidative stress in the process. Therefore, an elevation in blood glucose induces a reduction in insulin secretion, and diabetes becomes evident in the form of a vicious cycle that causes blood glucose elevation. Furthermore, as the oxidative stress status becomes chronic, the pancreatic B cells become apoptotic, leading to a decline in the number of functional pancreatic B cell 4. Insulin resistance involves important factors such as tumor necrosis factor (TNF)-α and adiponectin, which is secreted from fat cells and is one of the causes of type 2 diabetes. While TNF-α decreases the effect of insulin 5, adiponectin improves glucose tolerance 6.

Dermopathy is one of the symptoms of diabetes. As a result of dermopathy, skin dryness occurs owing to the decline in sweat gland activity by dysautonomia and/or dehydration as a result of hyperglycemia. Because skin barrier function declines following dryness, resistance to bacterial irruption decreases, and the risk of skin infection increases 7. The symptoms of diabetic dermopathy include hot flush, focal sudorrhea, carotenoid pigmentation, and acrocyanosis 8. Diabetes greatly influences the immune system 9, 10, which may also be affected in diabetic dermopathy. Furthermore, activation of vascular endothelial cells and induction of coagulation reinforcement status may occur in diabetes and in dermopathy.

In this study, we confirmed the onset of the skin dryness in type 1 and type 2 diabetes and tried to examine the pathogenic mechanism. We also evaluated skin dryness according to the difference in diabetes types by using streptozotocin (STZ)-induced diabetes mice as type 1 diabetes model mice, N-STZ induced diabetes model mice (non-obesity type), and KK-Ay/TaJcl mice (obesity type) as type 2 diabetes model mice.

## Materials and Methods

### Animal experiments

Specific-pathogen-free (SPF) C57BL/6N male mice (10-week-old) (CLEA Japan, Inc., Meguro-ku, Tokyo Japan), SPF C57BL/6N male mice (2-day-old) (CLEA Japan, Inc.), and SPF KK-Ay/TaJcl male mice (14-week-old) (CLEA Japan, Inc.) were used. They were housed in individual cages in an air-conditional room at 23 ± 1°C under SPF conditions with a 12 h light/12 h dark cycle and free access to drinking water and a pelleted basal diet. At the time of the last experiment, a blood glucose level of 300 mg/dl or more was considered as diabetes onset in the animal. And also C57BL/6N mice were used in the study, the same result was indicated on the other mouse strain (BALB/c and ICR mice) (data not shown). This study was performed in strict accordance with the recommendations of the Guide for the Care and Use of Laboratory Animals of Suzuka University of Medical Science (Approval number: 34). All surgeries were performed under pentobarbital anesthesia, and all efforts were implemented to minimize animal suffering.

### Study design

**Figure 1:**
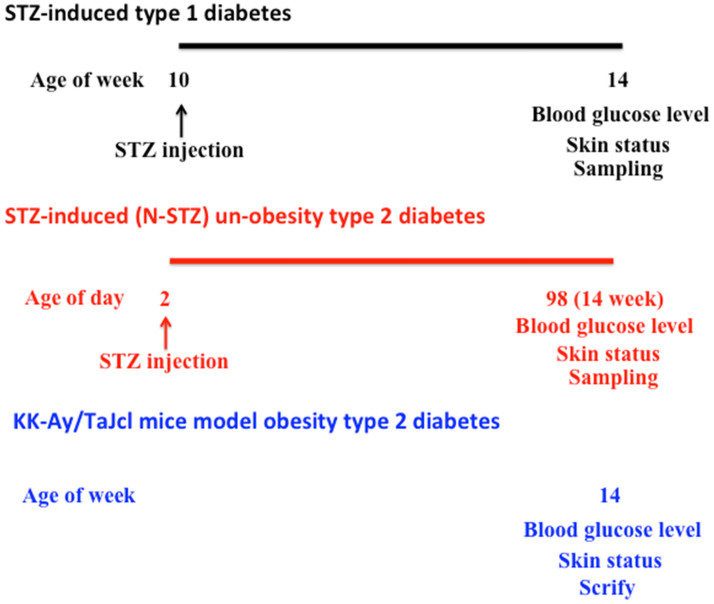
Schematic of the study-procedure.

We have summarized the experimental design in Figure 1.

### Type 1 diabetes 11-14

The mice were quarantined for the first 7 days and then randomized according to bodyweight into 2 groups (experimental and control groups), 10 mice per group. In the STZ group, mice were treated with STZ (250 mg/kg) (Sigma-Aldrich, Darmstadt, HE, Germany) through a single intraperitoneal injection. STZ is an antibiotic extracted from *Streptomyces achromogenes* and is an N-nitroso derivative of glucose (15). STZ causes a quick and irreversible necrocytosis of the pancreatic B cell 16. Four weeks after injection, we started to measure the body weight, amount of drinking water per day, urine volume per day, and blood glucose level. Control mice were untreated throughout the duration of the experiment.

### Type 2 diabetes (non-obesity type) 17-19

We categorized the newborn mice into 2 groups (N-STZ and control groups), 10 mice per group. STZ mice in the N-STZ group were administered a single intraperitoneal injection of STZ (250 mg/kg) on day 2 after birth. Starting at 14 weeks after the injection, we measured the body weight, amount of drinking water per day, urine volume per day, and blood glucose level. Control mice were untreated throughout the duration of the experiment.

### Type 2 diabetes (obesity type) 20, 21

KK-Ay/TaJcl mice are noninsulin-dependent-diabetes model mice, in which several genes are affected. The mice were quarantined for the first 7 days, and then divided into 2 groups (KK-Ay/TaJcl mice and control groups), 10 mice per group. Four weeks after injection, we measured the body weight, amount of water intake per day, urine volume per day, and blood glucose level. As a control, C57BL/6N mice of the same age were used.

### Measurement of transepidermal water loss (TEWL) and skin hydration

TEWL and skin hydration levels of the dorsal skin were measured on the final day of the examination, according to previously described methods 22.

### Preparation and staining of the dorsal skin

We obtained dorsal skin samples on the final day of examination. The skin samples were fixed in phosphate-buffered paraformaldehyde (4%), embedded in frozen Tissue-Tek, an OCT compound, and cut into 5 µm sections. The sections were stained with hematoxylin-eosin (HE) in accordance with the established procedures to enable histological analysis of the skin. To evaluate collagen expression, samples were stained by the Masson trichrome technique (trichrome stain kit [modified Masson's]; ScyTec Laboratories, Inc., Logan, UT, USA) 23. Additionally, skin specimens were stained with toluidine blue to visualize mast cells, and the stained skin tissues were microscopically evaluated following conventional procedures. Thereafter, the specimens were stained using an antibody for immunohistological analysis, as described previously 22. The skin specimens were incubated with a rabbit polyclonal anti-collagen IV (1:1000; Abcam) primary antibody. The specimens were subsequently incubated with fluorescein isothiocyanate-conjugated anti-rabbit secondary antibody (1:30; Dako Cytomation, Glostrup, Denmark). The expression of collagen IV was evaluated immunohistochemically by fluorescence microscopy.

### Quantification of matrix metalloproteinase (MMP)-1, MMP-2, MMP-9, histamine, hyaluronidase, hyaluronic acid, interleukin (IL)-6, TNF-α, and advanced glycation end products (AGEs) using an enzyme-linked immunosorbent assay (ELISA)

We extracted blood samples from the heart of the test mice on the final day of experiments. The plasma levels of MMP-1, MMP-2, MMP-9, histamine, hyaluronidase, hyaluronic acid, IL-6, TNF-α, and AGEs were determined using commercial ELISA kits (MMP-1: MyBioSource, San Diego, CA, USA; MMP-2, MMP-9, hyaluronic acid, and TNF-α: R&D Systems, Minneapolis, MN, USA; histamine: Bertin Pharm., Montigny-le-Bretonneux, France; hyaluronidase: AB Clonal Inc., Tokyo, Japan; IL-6: Proteintech, Rosemont, IL, USA; and AGEs: OxiSelect AGE connection ELISA kit, Cell Biolabs Inc., San Diego, CA, USA), according to the instructions of the respective manufacturers.

### Statistical analysis

All data are presented as the mean ± standard deviation. Results were statistically analyzed using Microsoft Excel 2010, with one-way analysis of variance, followed by Tukey's post-hoc test in SPSS software version 20 (IBM, Aemonk, NY, USA). Differences were considered statistically significant at *P* < 0.05.

## Results

### Effect of diabetes on body weight, water intake, urine production, and blood glucose levels

**Figure 2:**
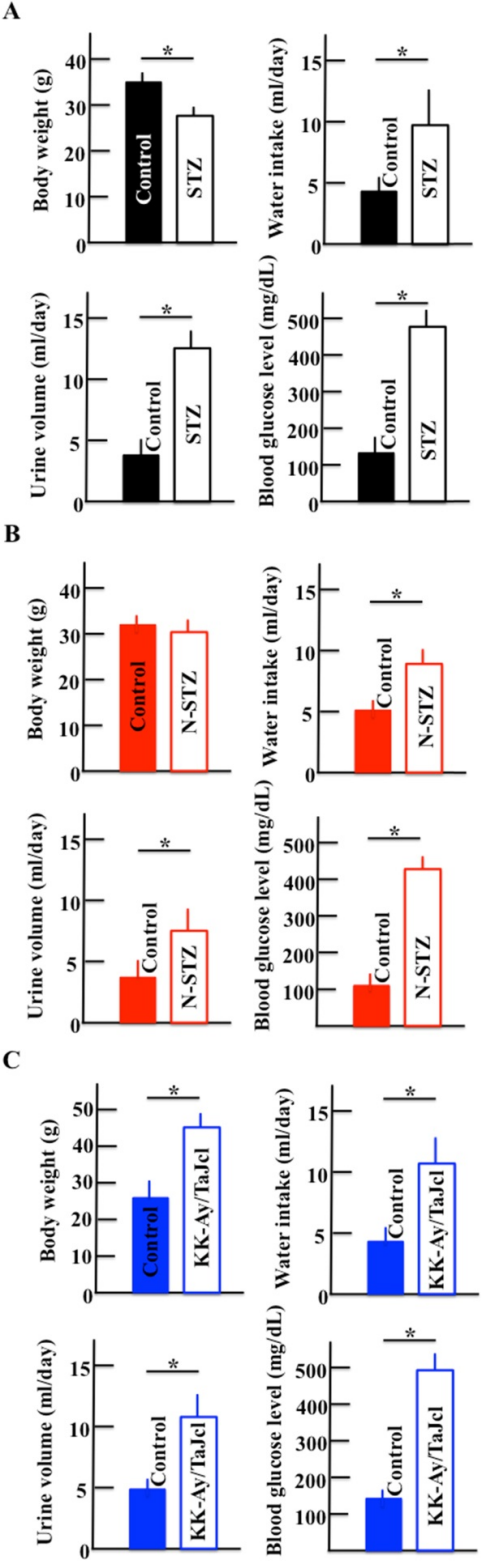
Effect of diabetes on body weight, water intake, urine volume, and blood glucose levels in mice. Type 1 diabetes model mice (A: streptozotocin [STZ]), non-obesity type 2 model mice (B: N-STZ), and obesity type 2 model mice (C: KK-Ay/TaJcl). The values are expressed as the mean ± standard deviation (SD) of six animals. *P < 0.05.

Figure 2 shows the body weights, water intake, urine production, and blood glucose levels of STZ (Fig. 2A) and N-STZ (Fig. 2B) or KK-Ay/TaJcl mice (Fig. 2C). The body weight was lower in the STZ mice and higher in the KK-Ay/TaJcl mice than in the control mice. The bodyweight of N-STZ mice was not different from that of the control. The water intake, urine production, and glucose levels were also found to be higher in all types of diabetic mice than in the control.

### Effect of diabetes on TEWL and skin hydration

**Figure 3:**
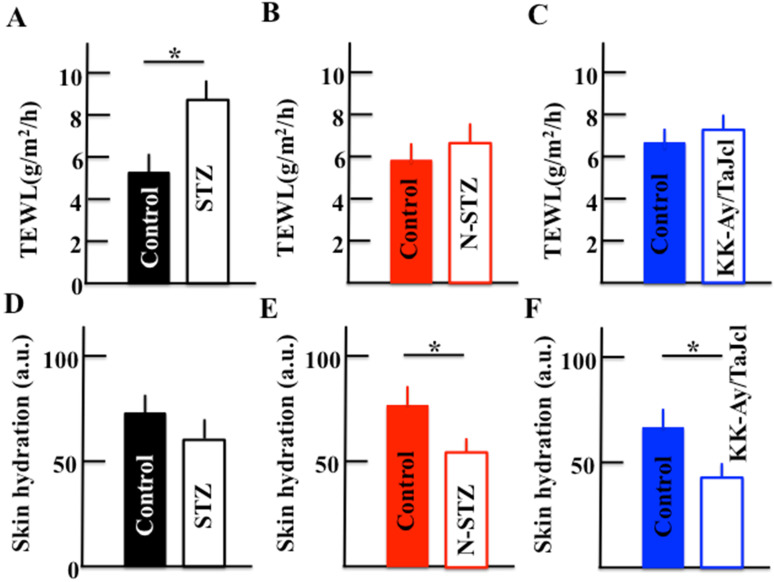
Effect of diabetes on transepidermal water loss (TEWL) and skin hydration in mice. Type 1 diabetes model mice (A, D: streptozotocin [STZ]), non-obesity type 2 model mice (B, E: N-STZ), and obesity type 2 model mice (C, F: KK-Ay/TaJcl). The values are expressed as the mean ± standard deviation (SD) of six animals. *P < 0.05.

During an initial investigation, we measured TEWL in control and diabetes mice models in triplicate and observed that these were the same in each group (data not shown); these results formed the basis for the current study in which we examined both TEWL and skin hydration levels in the stratum corneum in the control and diabetes model mice (Fig. 3). The TEWL levels were higher in the STZ mice than in the control mice (Fig. 3A), while the levels were similar in the N-STZ, KK-Ay/TaJcl, and control mice (Fig. 3B and 3C). In contrast, the skin hydration levels were lower in all diabetes model mice than in the control mice (Fig. 3D, 3E and 3F).

### Effect of diabetes on the plasma levels of AGEs

**Figure 4:**
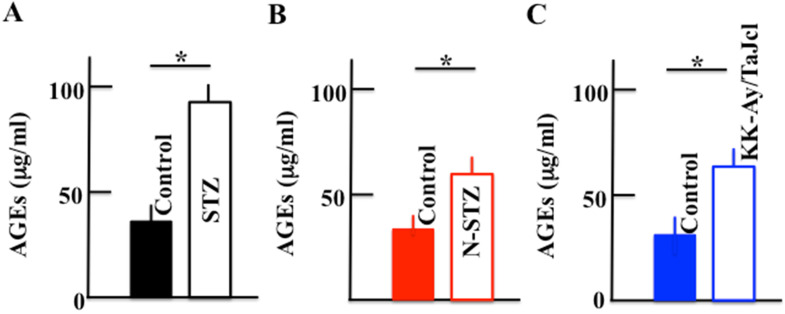
Effect of diabetes on the plasma level of advanced glycation end products (AGEs) in mice. Type 1 diabetes model mice (A: streptozotocin [STZ]), non-obesity type 2 model mice (B: N-STZ), and obesity type 2 model mice (C: KK-Ay/TaJcl). The values are expressed as the mean ± standard deviation (SD) of six animals. *P < 0.05.

The plasma levels of AGEs increased in the diabetes model mice compared to those in the control mice, with a significant increase in the STZ, N-STZ, and KK-Ay/TaJcl mice (Fig. 4).

### Effect of diabetes on total skin collagen

**Figure 5:**
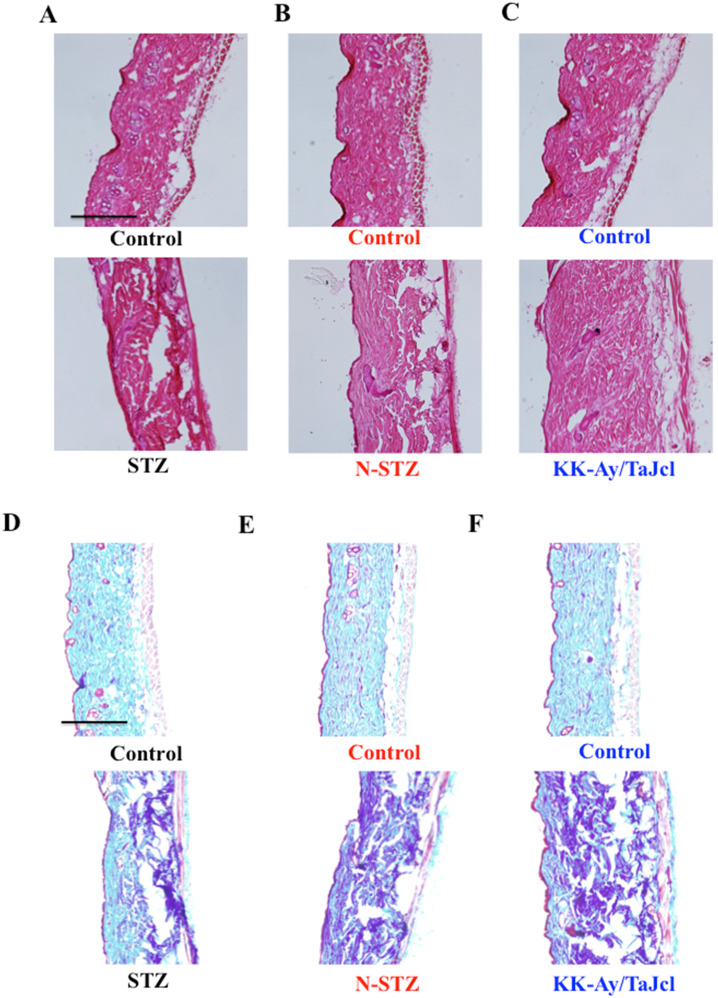
Effects of diabetes on collagen expression in the dorsal skin of mice. Histological analysis of skin sections, hematoxylin-eosin (HE) staining, and Masson-trichrome staining. Type 1 diabetes model mice (A, D: streptozotocin [STZ]), non-obesity type 2 model mice (B, E: N-STZ), and obesity type 2 model mice (C, F: KK-Ay/TaJcl). The data show 1 representative experiment performed on 10 animals. Scale bar = 100 µm.

The dermal expression of collagen decreased in the diabetes model mice compared to that in the control mice (Fig. 5), with a remarkable decrease in the N-STZ and KK-Ay/TaJcl mice compared to that in the control mice.

### Effect of diabetes on skin collagen type IV

**Figure 6:**
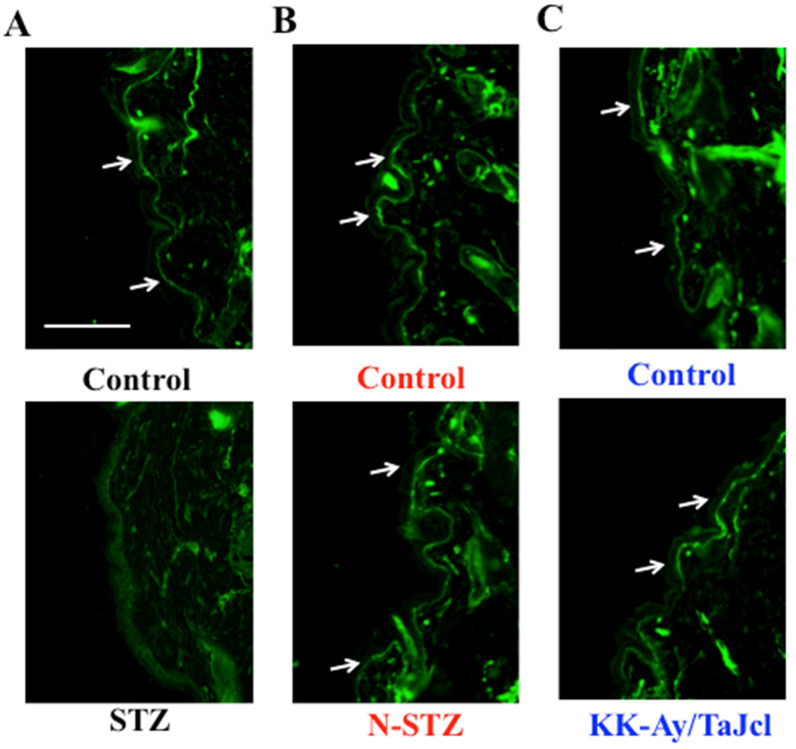
Effects of diabetes on collagen IV expression in the dorsal skin of mice. Type 1 diabetes model mice (A: streptozotocin [STZ]), non-obesity type 2 model mice (B: N-STZ), and obesity type 2 model mice (C: KK-Ay/TaJcl). Arrows indicate the collagen IV. The data show 1 representative experiment performed on 10 animals. Scale bar = 100 µm.

Next, we measured collagen type IV expression on the basement membrane of the epidermis. Collagen IV expression was lower in the STZ mice than in the control mice, while it was nearly similar in the N-STZ, KK-Ay/TaJcl, and control mice (Fig. 6).

### Effect of diabetes on the plasma levels of MMP-1, MMP-2, and MMP-9

**Figure 7:**
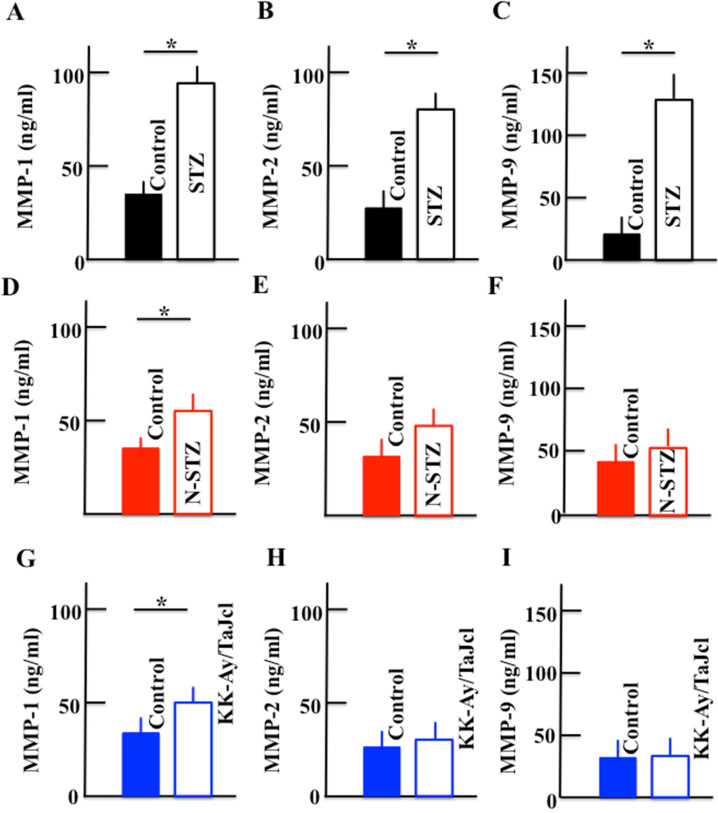
Effect of diabetes on the plasma levels of MMP-1, MMP-2, and MMP-9 in mice. Type 1 diabetes model mice (A, B, C: streptozotocin [STZ]), non-obesity type 2 model mice (D, E, F: N-STZ), and obesity type 2 model mice (G, H, I: KK-Ay/TaJcl). The values are expressed as the mean ± standard deviation (SD) of six animals. *P < 0.05.

The plasma levels of MMP-1, MMP-2, and MMP-9 increased in the STZ mice. In N-STZ and KK-Ay/TaJcl mice, the plasma level of MMP-1 increased, while the levels of MMP-2 and MMP-9 were not different from those of the control mice (Fig. 7).

### Effect of diabetes on the expression of skin mast cell and plasma levels of histamine and hyaluronidase

**Figure 8:**
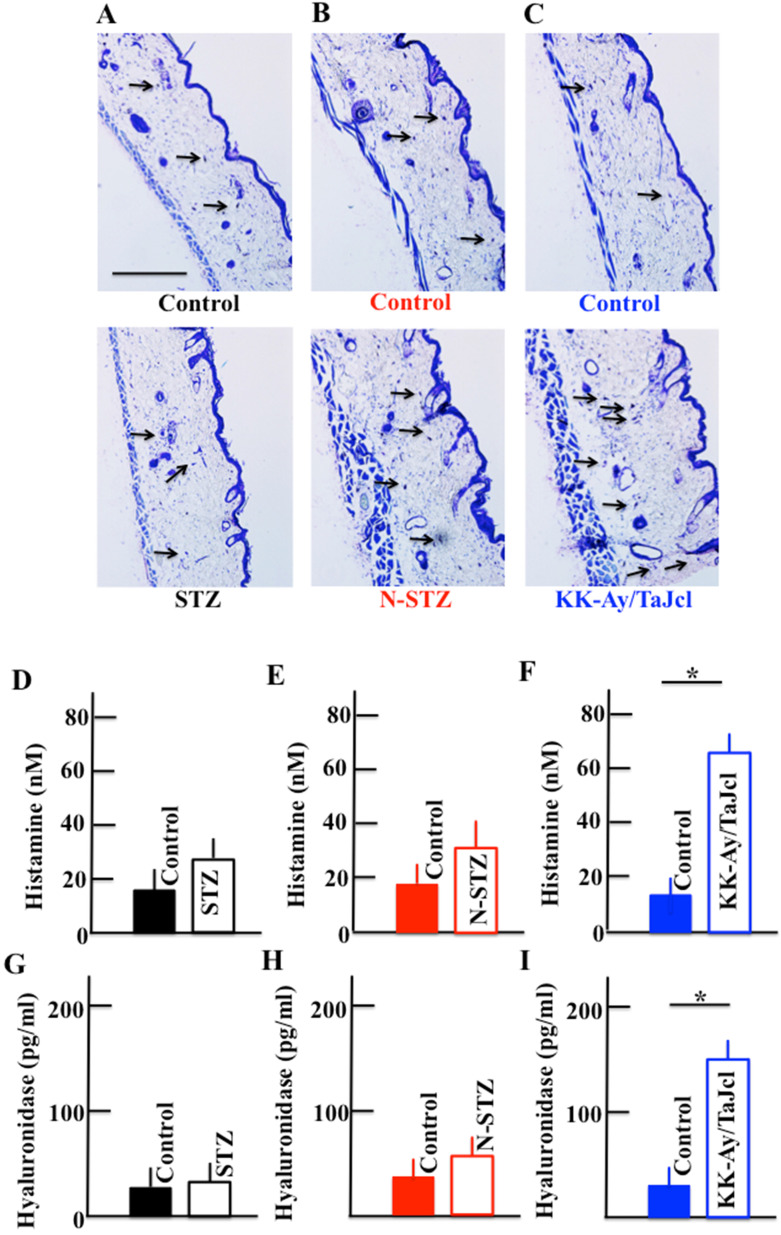
Effect of diabetes on the expression of mast cells in the dorsal skin and the plasma levels of histamine and hyaluronidase in mice. Type 1 diabetes model mice (A, D, G: streptozotocin [STZ]), non-obesity type 2 model mice (B, E, H: N-STZ), and obesity type 2 model mice (C, F, I: KK-Ay/TaJcl). Arrows indicate collagen IV. The data show 1 representative experiment performed on 10 animals. Scale bar = 100 µm. The values are expressed as the mean ± standard deviation (SD) of six animals. *P < 0.05.

The expression of mast cells increased in the skin of the N-STZ and KK-Ay/TaJcl mice compared to that in in the skin of the control mice (Fig. 8A, 8B and 8C), with a remarkable increase in the KK-Ay/TaJcl mice. The level of mast cells was similar in the skin of the STZ and control mice. Further, the plasma levels of histamine and hyaluronidase increased in the KK-Ay/TaJcl mice compared with those in the control mice (Fig. 8F and 8I). However, in the STZ and N-STZ mice, the levels of histamine and hyaluronidase were nearly similar to the levels in the control mice (Fig. 8D, 8E, 8G and 8H).

### Effect of diabetes on the plasma hyaluronic acid level

**Figure 9:**
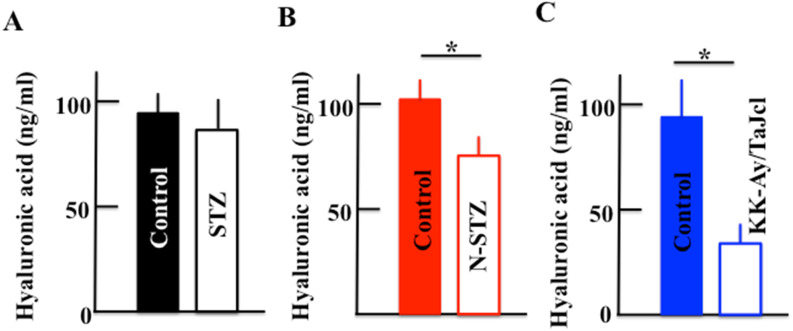
Effect of diabetes on the plasma level of hyaluronic acid in mice. Type 1 diabetes model mice (A: streptozotocin [STZ]), non-obesity type 2 model mice (B: N-STZ), and obesity type 2 model mice (C: KK-Ay/TaJcl). The values are expressed as the mean ± standard deviation (SD) of six animals. *P < 0.05.

The plasma level of hyaluronic acid decreased in the N-STZ and KK-Ay/TaJcl mice, although, in the STZ mice, the level was nearly similar to that in the control mice (Fig. 9A). The decrease of hyaluronic acid was the most remarkable in the KK-Ay/TaJcl mice compared to that in the control (Fig. 9C).

### Effect of diabetes on the plasma levels of IL-6 and TNF-α

**Figure 10:**
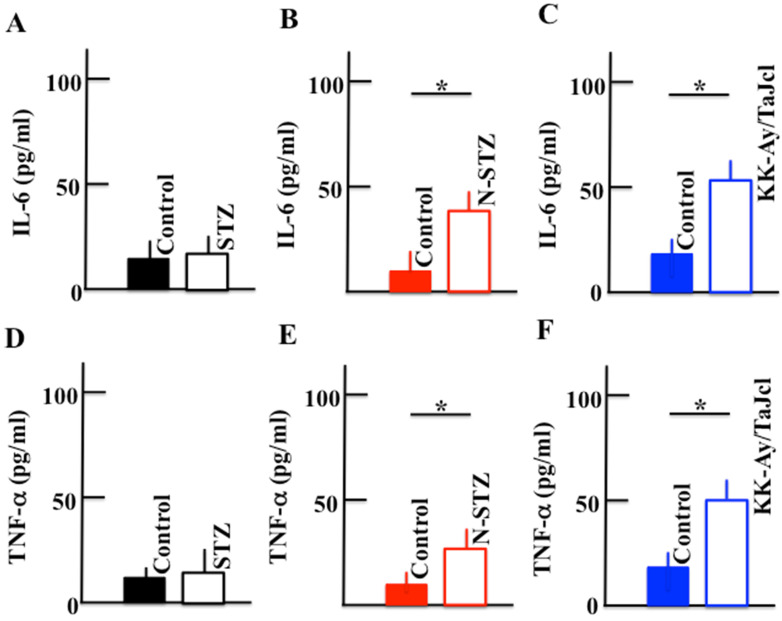
Effect of diabetes on the plasma levels of IL-6 and TNF-α in the mice. Type 1 diabetes model mice (A, D: streptozotocin [STZ]), non-obesity type 2 model mice (B, E: N-STZ), and obesity type 2 model mice (C, F: KK-Ay/TaJcl). The values are expressed as the mean ± standard deviation (SD) of six animals. *P < 0.05.

The plasma levels of IL-6 and TNF-α increased in the N-STZ and KK-Ay/TaJcl mice, while their levels were nearly similar in the STZ and control mice (Fig. 10).

## Discussion

In this study, the STZ, N-STZ, and KK-Ay/TaJcl mice were used to study skin dryness. The total collagen in the skin decreased in all mice models and decreased remarkably in the N-STZ and KK-Ay/TaJcl mice. Likewise, the collagen IV expression in the basement membrane decreased in the STZ mice, although the expression was nearly similar in the N-STZ, KK-Ay/TaJcl, and control mice. The plasma levels of MMP-1, MMP-2, and MMP-9 increased in the STZ mice, while only the level of MMP-1 increased in the N-STZ and KK-Ay/TaJcl mice compared to that in the control. The expression of skin mast cells and levels of histamine and hyaluronidase, which are released from the mast cells, increased remarkably in the KK-Ay/TaJcl mice. The level of hyaluronic acid decreased remarkably in the KK-Ay/TaJcl mice. In addition, although the levels of IL-6 and TNF-α increased in the N-STZ and KK-Ay/TaJcl mice, the levels were similar in the STZ and control mice.

Skin dryness was observed in both type 1 and type 2 diabetes model mice. However, the pathogenic mechanism of skin dryness in type 1 and type 2 diabetes model mice was different. In the type 1 diabetes mice, when diabetes develops, hyperglycemia occurs with the accumulation of AGEs. In a state of chronic hyperglycemia, AGEs accumulate in the circulating blood or in the tissue, causing organopathy 24. The AGEs accelerate the expression of the receptor for AGEs (RAGE) 25. This AGE-RAGE system induces transcription factor phosphorylation, which is mainly concerned with NF-κB through enhanced active oxygen production, mitogen-activated protein kinase activation, and activation of low-molecular-weight G protein by an intracellular. Consequently, the expression of MMP-9 is elevated 26. MMP-9 decomposes collagen IV in the basement membrane 27. Thus, in type 1 diabetes model mice, the increase in AGEs promotes the secretion of MMP-9 (Fig. 7), destroying the basement membrane (Fig. 6), increasing the loss of moisture through transpiration, and consequently, inducing skin dryness.

In obesity with type 2 diabetes, excessive macrophage infiltration between the enlarged fat cells and release of inflammatory cytokines occur, as TNF-α is abundantly secreted by fat cells in obese individuals 28. In obese individuals, chemokine-like monocyte chemoattractant protein-1 (MCP-1) is secreted, and M1 macrophages express excess C-C chemokine receptor 2, which is a receptor of MCP-1 induced in the fatty tissue 29. In the KK-Ay/TaJcl mice, the level of the inflammatory cytokines secreted from M1 macrophage remarkably increased compared to that in the control mice (Fig. 10). These inflammatory cytokines activate the mast cells, which subsequently release histamines 30-32 (Fig. 8). This in turn promotes the activity of hyaluronidase, a hydrolase of hyaluronic acid 33. The hyaluronidase then degrades hyaluronic acid, causing incomplete moisture retention and subsequent skin dryness.

In the N-STZ mice, the secretion of M1 macrophage from fat cells was nearly similar to that in the control mice. As a factor of skin dryness, we evaluated the AGE-RAGE system as observed in the type 1 diabetes mice. However, the increase in MMP-9 and degradation of collagen IV were not observed. Therefore, we hypothesize that skin dryness in type 2 diabetes occurs because the inflammatory cytokines and reactive oxygen species induced by the AGE-RAGE system decompose skin collagen 32; however, further studies are necessary to understand the mechanism involved.

## Conclusion

In this study, we observed skin dryness in both type 1 and type 2 diabetes conditions, with different mechanism of skin dryness in each type of diabetes. Skin dryness induces itchiness, causing various infective conditions, and poses problems to the diabetic individual. Therefore, understanding the mechanism of skin dryness will help in efficiently ameliorating the condition and improving the quality of living of the patient. In the current study, we used mouse models and our observations are not based on human examination. Therefore, it is necessary to perform clinical tests on humans to validate our findings.
